# Cannabinoids Modulate Light Signaling in ON-Sustained Retinal Ganglion Cells of the Mouse

**DOI:** 10.3389/fncir.2019.00037

**Published:** 2019-05-21

**Authors:** Terence Peter Middleton, Jin Yu Huang, Dario Alejandro Protti

**Affiliations:** ^1^Discipline of Physiology, The University of Sydney, Sydney, NSW, Australia; ^2^Bosch Institute, The University of Sydney, Sydney, NSW, Australia; ^3^Discipline of Biomedical Science, The University of Sydney, Sydney, NSW, Australia

**Keywords:** cannabinoids, synaptic modulation, receptive field, surround inhibition, area response function

## Abstract

The sole output of the retina to the brain is a signal that results from the integration of excitatory and inhibitory synaptic inputs at the level of retinal ganglion cells (RGCs). Endogenous cannabinoids (eCBs) are found throughout the central nervous system where they modulate synaptic excitability. Cannabinoid receptors and their ligands have been localized to most retinal neurons in mammals, yet their impact on retinal processing is not well known. Here, we set out to investigate the role of the cannabinoid system in retinal signaling using electrophysiological recordings from ON-sustained (ON-S) RGCs that displayed morphological and physiological signatures of ON alpha RGCs in dark adapted mouse retina. We studied the effect of the cannabinoid agonist WIN55212-2 and the inverse agonist AM251 on the spatial tuning of ON-S RGCs. WIN55212-2 significantly reduced their spontaneous spiking activity and responses to optimal spot size as well as altered their spatial tuning by reducing light driven excitatory and inhibitory inputs to RGCs. AM251 produced the opposite effect, increasing spontaneous spiking activity and peak response as well as increasing inhibitory and excitatory inputs. In addition, AM251 sharpened the spatial tuning of ON-S RGCs by increasing the inhibitory effect of the surround. These results demonstrate the presence of a functional cannabinergic system in the retina as well as sensitivity of ON-RGCs to cannabinoids. These results reveal a neuromodulatory system that can regulate the sensitivity and excitability of retinal synapses in a dynamic, activity dependent manner and that endocannabinoids may play a significant role in retinal processing.

## Introduction

Endocannabinoids (eCB) are potent modulators of synaptic transmission found throughout the central nervous system. ECBs control cell excitability via a localized short-range synaptic mechanism whereby they are released by a postsynaptic neuron in response to depolarisation and travel retrogradely to activate presynaptic cannabinoid receptors (*CB1R* and/or *CB2R*). The activation of CBRs, in turn, leads to a reduction in the release probability of neurotransmitter from presynaptic neurons (Castillo et al., 2012).

ECBs [arachidonoylethanolamine (AEA) and 2-arachidonoylglycerol (2-AG)] and their receptors have been localized to most retinal cells. CB1R was found in the inner and outer plexiform layers (IPL and OPL) of the monkey, mouse, human and other species (Straiker A. et al., 1999; Straiker A. J. et al., 1999; Yazulla et al., 1999, 2000). More recent studies showed CB1R and fatty acid amide hydrolase (FAAH), the enzyme responsible for degradation of AEA, on all retinal cells from early developmental stages, in rat and vervet monkey (Glaser et al., 2005; Zabouri et al., 2011a,b; Bouskila et al., 2012). CB2R and CB2R mRNA has also been detected in the retina (Lu et al., 2000; Lopez et al., 2011; Cottone et al., 2013). Moreover, a cannabinoid sensitive G-protein coupled receptor, GPR55, is also expressed on rod photoreceptors in the vervet monkey (Bouskila et al., 2013). Several reports indicate that cannabinoids have an active role in retinal processing in humans and other primates (Zobor et al., 2015; Bouskila et al., 2016) and it has been postulated that cannabinoids potentially improve night vision (Russo et al., 2004), suggesting that retinal cannabinoids are involved in dark adaptation mechanisms. Moreover, cannabis use has also been linked to delays in RGC signal transmission in humans (Schwitzer et al., 2017).

Cannabinoids were shown to modulate K^+^ and Ca^2+^ currents in the rods and cones of goldfish and tiger salamander (Fan and Yazulla, 2003; Straiker and Sullivan, 2003), an effect that is endogenously controlled by postsynaptic release of 2-AG from bipolar cells (Fan and Yazulla, 2007). This modulation has been shown to alter the kinetics of light responses in goldfish cones (Struik et al., 2006). In bipolar cells WIN55212-2, a cannabinoid receptor agonist, reduced L-type calcium currents (Straiker A. et al., 1999) and inhibited the delayed rectifier K^+^ current in tiger salamander (Yazulla et al., 2000). In addition, WIN55212-2 was shown to reduce the frequency of spontaneous inhibitory synaptic currents in cultured embryonic chick amacrine cells (Warrier and Wilson, 2007). Despite the widespread distribution of CBRs in the retina, the effects of both exo- and endo-cannabinoids in retinal function are poorly understood.

Retinal ganglion cells (RGCs) integrate excitatory and inhibitory synaptic inputs from bipolar and amacrine cells, respectively, and provide output signals from the retina to other CNS areas. A cannabinoid agonist has been shown to inhibit high voltage activated Ca^2+^ channels (Lalonde et al., 2006) and may influence the kinetics of evoked action potentials (Jiang et al., 2013). Additionally WIN55212-2 was shown to suppress K^+^ currents in RGCs independently of CB1R or CB2R (Zhang et al., 2013).

It has previously been shown that WIN55212-2 reduces the frequency of spontaneous excitatory and inhibitory synaptic currents (EPSCs and IPSCs) in mouse RGCs (Middleton and Protti, 2011). CB1R was also shown to modulate the frequency of IPSCs and EPSCs in RGCs via inhibition of L-type and T-type Ca^2+^ channels, respectively (Wang et al., 2016). More recently, eCBs were shown to modulate calcium influx into RGC via modulation of transient receptor potential vanilloid type 1 (TRPV1) and CB1R (Jo et al., 2017). Thus far, however, no study investigated the effects of cannabinoids on visually evoked responses of mammalian RGCs.

Here, we investigate the effects of WIN55212-2 and the CB1R inverse agonist AM251 on visually evoked responses of ON-sustained (ON-S) RGCs in the mouse and on their synaptic inputs. We find that activation of cannabinoid receptors reduces both spontaneous firing rate and the strength of visual-evoked responses as well as broadens the spatial tuning of ON-S RGCs. Using a CB1R inverse agonist, we reveal that in the retina there is basal activity of the cannabinergic system that modulates spontaneous firing rate, transmission of visual evoked signals and the receptive field organization of ON-S RGCs. Finally, we demonstrate that cannabinoids modify RGC responses and their receptive field properties by affecting the spatial modulation of excitatory and inhibitory synaptic conductances in different ways.

## Results

### A Cannabinoid Agonist Reduces Spontaneous Firing Rate and Alters Receptive Field Properties of ON-S RGCs

**FIGURE 1:**
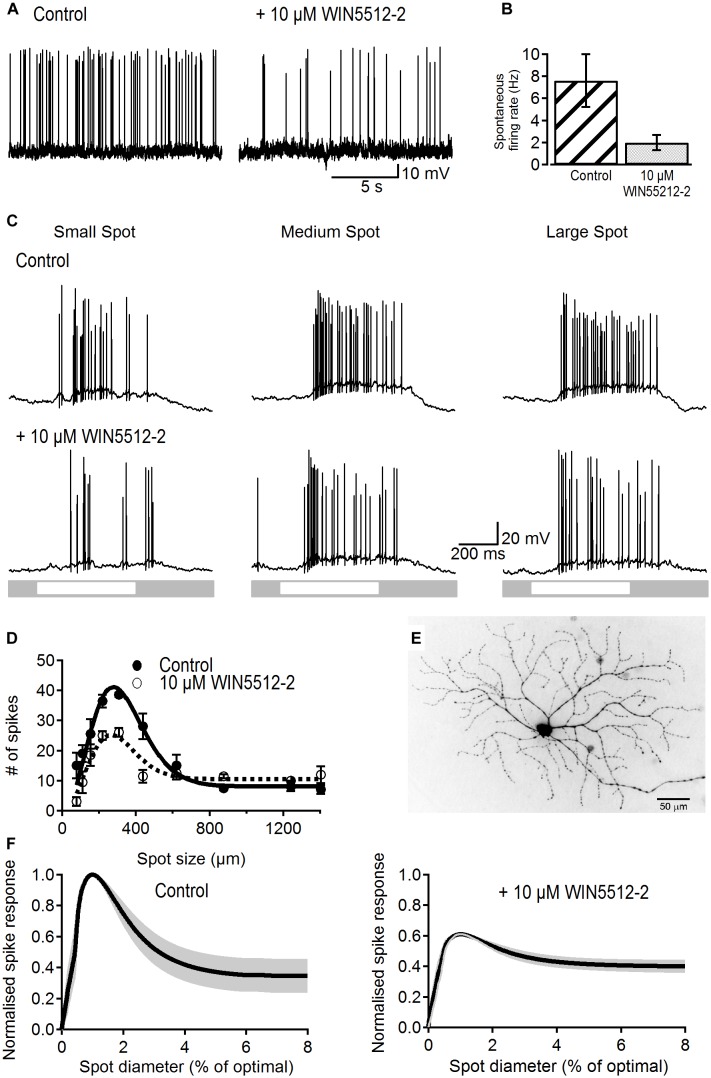
Effects of a cannabinoid agonist on spontaneous activity and receptive field properties of ON-S RGCs. **(A)** Representative traces showing the reducing effect of WIN55212-2 (10 μM) on spontaneous spike rate. **(B)** Bar plot showing that the spontaneous frequency of action potentials was significantly reduced in the presence of WIN55212-2 from 7.6 ± 2.38 to 2.0 ± 0.68 spikes/s (*p* < 0.05, *n* = 8). **(C)** Representative traces showing responses of an ON-S RGC to different sized spot stimuli. Stimulation of the cell’s receptive field with a small (154 μm) bright spot caused a response consisting of an increase in firing rate (first column). As the spot increases in size more of the receptive field is stimulated increasing the response. Stimulation of a larger area of the receptive field center causes a larger response (second column, spot size 308 μm). When the spot size increases further the antagonistic surround receptive field is activated and reduces the excitatory response (third column, spot size 1240 μm). After addition of a cannabinoid agonist WIN55212-2 (10 μM) the response to the light spot is decreased. **(D)** Number of spikes in response to spot stimuli of different sizes for a representative cell. The data is fitted to a Difference of Gaussians (DoG) function. Addition of a cannabinoid agonist lowered the peak response but also lowered the degree of surround inhibition that dampens the peak response observed at larger spot sizes. Symbols represent the average response to two stimulus presentations for each size, bars represent standard deviation. **(E)** Lucifer Yellow filled ON-S RGC that was treated with WIN55212-2. Scale bar = 50 μm. **(F)** Average curve fits from all cells tested with WIN55212-2 (*n* = 9) with the SEM shown in gray. Curves were normalized to the peak response as well as the stimulus size that elicited the peak response.

Spontaneous firing rate was recorded against background luminance from 20 dark-adapted ON-S RGCs. Cells were either treated with a cannabinoid receptor agonist or an inverse agonist and their changes in spike frequency recorded. Bath application of the cannabinoid agonist WIN55212-2 (10 μM) significantly reduced spontaneous firing rate from 7.6 ± 2.38 to 2.0 ± 0.68 Hz (*p* < 0.05, *n* = 8; Figure 1A,B) with no significant change in membrane potential (-65.3 ± 2.4 vs. -66.3 ± 2.4 mV, *p* > 0.2).

To test whether or not retinal signaling is also modulated by cannabinoids we investigated the effects of WIN55212-2 on visual-evoked responses and receptive field organization in ON-S RGCs as they display well characterized center-surround organization.

Spiking response to a small spot stimulus in control conditions typically produced a relatively weak response (Figure 1C, left top trace). Increasing spot size to a value that activates most of the receptive field center led to a maximal response whilst an even larger stimulus produced a weaker response due to recruitment of the inhibitory surround (Figure 1C middle and right top traces). Bath application of WIN55212-2 (10 μM) reduced the strength of light responses for all three sizes, demonstrating a modulatory influence of cannabinoids on RGC output (Figure 1C, bottom traces).

Figure 1D shows the area-response function of a representative RGC in control conditions and after WIN55212-2 application fitted to a DoG function (lines). WIN55212-2 (10 μM) reduced the peak response by 32% and reduced the suppression index from 75 to 42%. Figure 1E shows the morphology of the same RGC.

Figure 1F shows the average area–response function for all cells tested with WIN55212-2. Curves of individual cells were normalized to the peak response in control conditions and to the spot size that elicited the peak response. WIN55212-2 had no effect on the size of the receptive field center (average spot diameter in control conditions 277 ± 46 vs. 279 ± 43 μm after drug application; *p* > 0.9, *n* = 9). The peak response was significantly lowered in all cells by an average of 38 ± 8% (*p* < 0.05, *n* = 9). Furthermore, the SI was significantly reduced from 58 ± 10 to 35 ± 6% after application of 10 μM WIN55212-2(*p* < 0.05, *n* = 9). WIN55212-2 also significantly reduced the amplitude of the peak response of the light-evoked postsynaptic potential (LE-PSP) by 18% (13.4 ± 2.1 to 11 ± 1.7 mV, *p* < 0.05, *n* = 9) and the SI by 10% (44 ± 8 to 34 ± 8%, *p* < 0.05, *n* = 9, data not shown).

### Basal Cannabinergic Activity Modulates Response Strength and Shapes Spatial Tuning of ON-S RGCs as Revealed by a Cannabinoid Inverse Agonist

The effects exerted by the exogenous cannabinoid agonist WIN55212-2 on response strength and receptive field properties revealed sensitivity to cannabinoids, suggesting that endocannabinoids may have a physiological role. To test whether or not eCBs exert a modulatory effect in retinal signaling in physiological conditions we next examined the effect of the cannabinoid receptor inverse agonist AM251.

**FIGURE 2:**
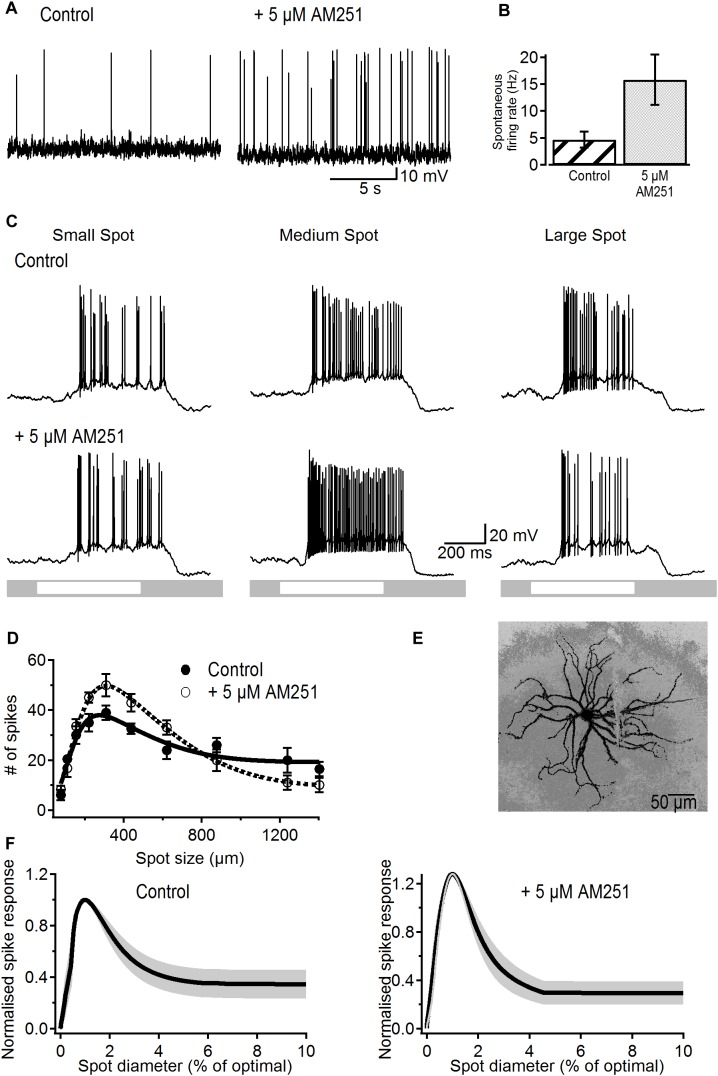
Effects of the CB1R inverse agonist on spontaneous activity and receptive field properties of ON-S RGCs. **(A)** Representative traces showing the increasing effect of AM251 (5 μM) on spontaneous firing rate. **(B)** Bar plot showing the significant increase in spontaneous firing rate induced by AM251 from 4.7 ± 1.5 to 15.8 ± 4.7 spikes/s (*p* < 0.05, *n* = 12). **(C)** A response from a representative cell to different sized spot stimuli. Small, medium, and large spot sizes were 154, 220, and 1240 μm, respectively. After addition of a CB1R inverse agonist AM251 (5 μM) the response to the light spot is increased. **(D)** Number of spikes in response to spot stimuli of different sizes for a representative cell. The data is fitted to a DoG function. Addition of AM251 increased the peak response but also increased the degree of surround inhibition that dampens the peak response observed at larger spot sizes. Symbols represent the average response to two stimulus presentations for each size, bars represent standard deviation. **(E)** Lucifer Yellow filled ON-S RGC that was treated with AM251. Scale bar = 50 μm. **(F)** Average curve fits from all cells tested with AM251 (*n* = 6) with the SEM shown in gray. Curves were normalized to the peak response to the stimulus size that elicited peak response.

Bath application of 5 μM AM251 produced a significant increase in the rate of spontaneous action potentials from 4.7 ± 1.5 to 15.8 ± 4.7 Hz (*p* < 0.05, *n* = 12; Figure 2A,B), demonstrating the presence of basal cannabinergic activity in the retina that upon blockade by the inverse agonist results in the observed increase in spike frequency. This increase in firing rate occurred without any significant change in membrane potential (-63.9 ± 1 vs. -64.2 ± 1 mV, *p* > 0.8)

Figure 2C shows light-responses from a representative ON-S RGC before and after treatment with AM251 (5 μM). Light-evoked responses typically displayed size tuning and the characteristic center/surround receptive field organization in control conditions (Figure 2C). Bath application of AM251 greatly increased the strength of the peak response but reduced the magnitude of the response to the largest spot (Figure 2C, bottom right). This effect suggests that blockade of eCBs acting via CB1Rs increases the strength of excitation recruited by stimulation of the receptive field center as well as the strength of the inhibitory surround.

The area-response function of this cell confirms that AM251 produced a stronger center response and more surround inhibition in comparison to control responses (Figure 2D). In this cell AM251 increased the peak response by 28% and the SI by 22% (SI: 58% in control vs. 80% after AM251). Figure 2E shows the morphology of this representative RGC.

Figure 2F shows the average area–response function of all cells treated with AM251. Data was normalized to the peak response and to the spot size that elicited the peak response prior to drug application. The average size of the receptive field center was 305 ± 45 μm in control conditions and 293 ± 34 μm after application of AM251 (*p* > 0.6, *n* = 6), indicating that AM251 had no effect on the center receptive field size. The peak spike response was significantly increased in all cells by an average of 28 ± 15% (*p* < 0.05, *n* = 6). Furthermore, the SI of the spike response was significantly increased from 65 ± 10 to 84 ± 7% after application of 5 μM AM251 (*p* < 0.05, *n* = 6). AM251 caused a small and non-significant increase of 16.6% on the peak amplitude of the LE-PSP (16.8 ± 2.7 to 19.6 ± 3.9 mV, *n* = 6, *p* > 0.5) and on SI (58 ± 13 to 68 ± 12%, *n* = 6; *p* > 0.25, data not shown).

### Cannabinoids Modify the Inputs to ON-S Retinal Ganglion Cells

**FIGURE 3:**
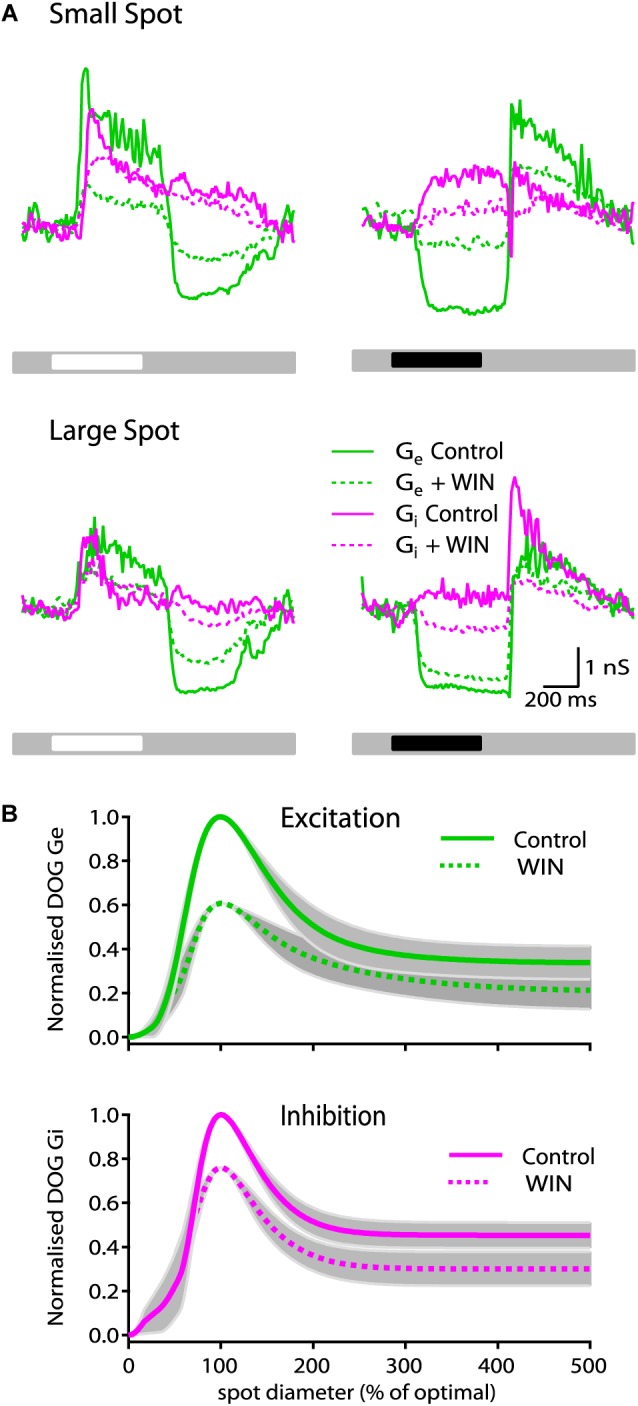
WIN55212-2 reduces the strength of synaptic conductances in ON-S RGCs. **(A)** Representative ON-S cell showing the excitatory (G_exc_) and inhibitory (G_inh_) conductance in response to small (266 μm) bright (top left) and dark (top right) spot stimuli. Addition of WIN55212-2 (5 μM) reduced both the G_exc_ (57%) and the G_inh_ (17%) response to the light stimulus. Bottom traces show responses of the same cell to large (1275 μm) bright (left) and dark (right) stimuli. **(B)** Top plot illustrates the receptive field profile of G_exc_ represented by the mean DoG curve fits for all cells. Addition of WIN55212-2 (10 μM) reduced the peak response by 39 ± 15% and the surround inhibition by 9% (SI: 61 ± 6 to 52 ± 7%, *p* < 0.05, *n* = 5). Bottom plot shows that addition of WIN55212-2 (5 μM) reduced the peak G_inh_ response by 23 ± 17% (*p* < 0.05, *n* = 5) and the surround inhibition by 2% (SI: 52 ± 5 to 50 ± 9, *n* = 5).

Under mean background illumination ON-S RGCs receive tonic excitation from bipolar cells and stimulation with a bright spot typically provokes an increase in excitatory and inhibitory inputs that reach peak amplitude within ∼100 ms of stimulus onset and then decay to a constant value (Figure 3A, see also van Wyk et al., 2009). At stimulus offset the excitatory input typically decreases below the mean tonic input and then it returns to the constant tonic input levels within ∼500 ms. Inhibition at stimulus offset is typically smaller in magnitude. To elucidate the synaptic mechanisms responsible for the effects of the cannabinoid agonist and inverse agonist on response strength and receptive field organization, we conducted whole-cell voltage clamp recordings and carried out conductance analysis to dissect the excitatory and inhibitory inputs generated by spots of different sizes.

Presentation of a 250 μm bright spot on a gray background produced an increase in both G_exc_ (green line) and G_inh_ (magenta line) which after ∼150 ms declined to a constant value (Figure 3A, left top traces). Bath application of WIN55212-2 (10 μM) reduced the strength of the response by 57% for G_exc_ (dotted green line) and 17% for G_inh_ (dotted magenta line) at stimulus onset. The magnitude of the change in G_exc_ at stimulus offset was also reduced by 48% whilst and G_inh_ at stimulus offset was only reduced by 17%. Across all cells tested there was no significant change in tonic conductance for G_exc_ and G_inh_ measurements (data not shown). WIN55212-2 also decreased the magnitude of the reduction in tonic excitatory conductance and of the increase in inhibitory conductance elicited by a dark spot and it reduced the increases in G_exc_ and G_inh_ that occur at stimulus offset (Figure 3A, right top traces). When bright and dark large diameter bright spots were used, WIN55212-2 also reduced the magnitude of changes in G_exc_ and G_inh_ but to a lesser extent than when small spots were used (Figure 3A, bottom traces).

The area–response functions of G_exc_ and G_inh_ for individual RGCs was characterized by finding the best predictions of a DoG model of the receptive field. From these fits we extracted the spatial dimensions of the center and surround for each conductance, and an index of spatial tuning (SI). Figure 3B (top plot) shows the spatial tuning curve of G_exc_ for all cells calculated by averaging the DoG fits of each cell that were previously normalized to their control response amplitude and optimal size.

Across all cells tested, WIN55212-2 (10 μM) caused a reduction in the strength of light evoked G_exc_, with the peak response reduced by 39 ± 15% (*p* < 0.05, *n* = 5 cells). In addition, we observed a significant reduction of 9% (SI: 61 ± 6 to 52 ± 7, *p* < 0.05, *n* = 5) in the suppression index of G_exc_. WIN55212-2 did not modify the center radius of G_exc_ (263 ± 28 μm in control conditions vs. 275 ± 33 μm after bath application of WIN55212-2, *n* = 5 cells), consistent with the observations in spike responses. Figure 3B (bottom plot) shows the average receptive field-tuning curve for inhibitory conductance. Drug application reduced the peak light-evoked G_inh_ response by 23 ± 17% (*p* < 0.05, *n* = 5). However, the SI of G_inh_ was not significantly changed showing only a small reduction of 2% (SI: 52 ± 5 to 50 ± 9, *n* = 5). The center radius of G_inh_ did not change after application of WIN55212-2 (257 ± 48 in control conditions versus 241 ± 32 following bath application of WIN55212-2, *n* = 5 cells).

### A Cannabinoid Inverse Agonist Enhances Both Excitatory and Inhibitory Light-Evoked Synaptic Conductances

**FIGURE 4:**
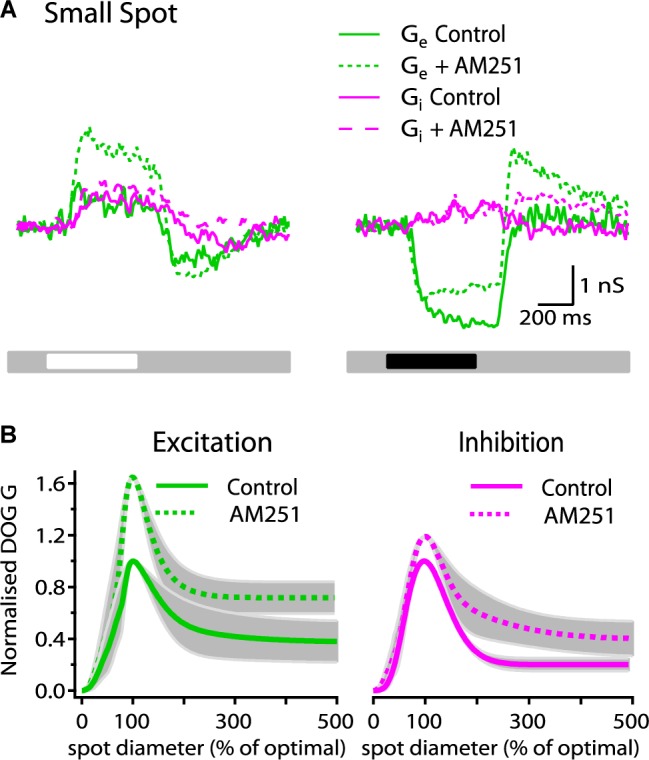
AM251 increases the strength of synaptic conductances in ON-S RGCs. **(A)** Representative ON-S cell showing the excitatory (G_exc_) and inhibitory (G_inh_) conductance in response to small (266 μm) bright and dark stimuli. Addition of AM251 (5 μM) increased both the G_exc_ (75%) and the G_inh_ (14%) response to the bright stimulus. AM251 decreased the reduction in tonic excitation at the onset of a dark spot and increased the change in Gexc at the offset of a dark spot. **(B)** Receptive field profiles of G_exc_ and G_inh_ represented by the mean DoG curve fits for all cells. AM251 (5 μM) increased the peak response of G_exc_ by 69 ± 21% (*p* < 0.05, *n* = 4) and reduced surround inhibition by 4% (SI: 61 ± 16 to 57 ± 12%, *n* = 4). Addition of AM251 (5 μM) increased the peak G_inh_ response by 18 ± 21% (*p* < 0.05, *n* = 4) and the surround inhibition by 14% (SI: 80 ± 5 to 66 ± 13%, *n* = 4).

To determine whether or not the effect of AM251 on spike response and receptive field organization is due to its influence on excitatory and/or inhibitory inputs, we studied the effect of AM251 (5 μM) on the magnitude and spatial organization of excitatory and inhibitory synaptic inputs. Figure 4A shows the excitatory and inhibitory conductance for a representative ON-S cell before and after bath application of AM251 (5 μM). AM251 had a strong effect on the light-evoked response, increasing both the peak G_exc_ (75%) and G_inh_ (14%). Across all cells tested, AM251 increased the peak G_exc_ by 69 ± 21% (*p* < 0.05, *n* = 4) and reduced the SI of G_exc_ by 4% (61 ± 16% in control conditions vs. 57 ± 12%, *n* = 4). AM251 also produced a decrease in the reduction in tonic excitation and did not affect the inhibitory conductance produced at the onset of a small, dark spot and produced a large increase in G_exc_ and a small increase in G_inh_ at stimulus offset (Figure 4A, right traces). Figure 4B shows the average DoG curves. The G_inh_ response was increased by 18 ± 21% (*p* < 0.05, *n* = 4) and the suppression index of G_inh_ inhibition was reduced by 14% (SI: 80 ± 5 to 66 ± 13, *n* = 4). AM251 had no effect on the radius of the center of G_exc_ (278 ± 35 to 293 ± 27 μm, *n* = 4 cells). The center radius of G_inh_ was not modified after application of AM251 (241 ± 31 in control conditions versus 259 ± 21 after bath application of AM251, *n* = 4 cells).

These results demonstrate the existence of basal cannabinergic activity that modulates the balance of excitatory and inhibitory inputs onto RGCs. This modulatory effect regulates not only the overall response strength but it also shapes the receptive field surround of the excitatory and inhibitory conductances and thus modifies the spatial tuning of ON-S RGCs. Given the uniform architecture of the cannabinoid signaling system throughout the retina it is likely that other RGC types are subject to similar modulatory mechanisms.

## Discussion

Our results demonstrate that cannabinoids modulate the transmission of visual-evoked responses by ON-S RGCs. Moreover, our data shows that in the retina there is a functional cannabinergic system that modulates the strength of the transmission of visual signals by ON-S RGCs as well as their spatial tuning. Thus, endocannabinoid signaling in the retina seems to play a similar role in the modulation of the strength of synaptic transmission as that described in other areas of the CNS (Chevaleyre et al., 2006).

The reduction in spontaneous firing rate induced by the cannabinoid receptor agonist WIN55212-2 is consistent with previous reports showing that WIN55212-2 reduced the frequency of excitatory and inhibitory spontaneous synaptic currents in mouse (Middleton and Protti, 2011) and rat (Wang et al., 2016) RGCs. The observed reduction in firing rate suggests that WIN55212-2 produced a shift in the balance of excitation and inhibition, more strongly affecting the excitatory input onto ON-RGCs. A recent report found that WIN55212-2 did not have any effect on spontaneous firing rate in rat RGCs (Jiang et al., 2013). Their recording conditions, however, differed from our study in that recordings were obtained indistinctly from different types of RGCs in retinal slices in light-adapted conditions. Interestingly, we found that the CB1R inverse agonist AM251 showed the opposite effect leading to an increase in firing rate. This is, as far as we know, the first physiological evidence of basal cannabinergic activity in the mammalian retina that modulates RGC output to the rest of the brain. Assuming that the retinal cannabinergic system functions in a similar way to the endocannabinoid system in other brain areas, it can be postulated that an endocannabinoid “tone” is constitutively active in proportion to the activity of ON-S RGCs and that endocannabinoids travel retrogradely to activate cannabinoid receptors on amacrine and bipolar cells and lower their release probability. Alternatively, the effects of AM251 can be explained by constitutive activity of cannabinoid receptors independently of their activation by endocannabinoids. Although this is a possible scenario, a currently accepted interpretation of the effects of inverse agonists is that they act by blocking endogenously produced endocannabinoids that provide autocrine or paracrine stimulation of CB1 receptors, giving the appearance of constitutive activity (Howlett et al., 2011). Experiments blocking the synthetic enzymes of endocannabinoids (Farkas et al., 2010) or chelating postsynaptic calcium (Hentges et al., 2005) could further distinguish whether the inverse agonist acts by blocking a constitutively active receptor or by displacing an endogenous cannabinoid that provides a basal tone.

Although the presence of an endogenous cannabinoid tone in the mammalian retina suggests a role in retinal processing, its physiological effects on the retinal circuit had not been described thus far other than at the cellular level using strong stimulation protocols, which are not regarded as physiological (Diana and Marty, 2004). Wang et al. (2016) demonstrated that depolarization-induced suppression of inhibition, a well described short-term plasticity phenomenon in cerebellum and hippocampus, also takes place in RGCs. Application of depolarizing pulses to RGCs led to a suppression of their mIPSCs and this effect was eliminated by a CB1R antagonist, suggesting that eCBs are indeed released from RGCs in an activity-dependent manner. We probed the effects of cannabinoid receptor modulation on the response strength of ON-S RGCs and showed that the reduction in peak spike response induced by WIN55212-2 was the result of a reduction in the amplitude of the peak light-evoked postsynaptic potential. This effect may be due to: (1) a decrease in excitation, (2) an increase in inhibition, (3) a decrease in excitation and an increase in inhibition or (4) a decrease in excitation and a decrease in inhibition. Conductance analysis revealed that it was in fact owing to a greater decrease in the strength of the light-evoked excitatory synaptic inputs in relation to a reduction in inhibitory inputs, thus leading to an overall decrease in net excitation. Interestingly, a recent study on amphibian RGCs also found that cannabinoid receptor activation produced a reduction in visually evoked excitatory and inhibitory synaptic inputs in amphibian RGCs (Miraucourt et al., 2016). However, in contrast to what we found, this study, also reported that activation of CB1Rs hyperpolarized resting potential by inhibiting the Na^+^-K^+^-2Cl^-^ co-transporter 1 (NKCC1); hyperpolarization would lead to removal of inactivation of voltage-gated sodium channels and therefore enhance RGC excitability. Although we cannot rule out a direct effect of cannabinoids on the Na^+^-K^+^-2Cl^-^ co-transporter 1 in RGCs, if WIN55212-2 were to induce a shift of -3.5 mV in the reversal potential of chloride (as reported by Miraucourt et al., 2016), modeling of such a change predicts that it would result in an increase of only 2.3% in G_exc_ and a reduction of 5% in G_inh_. Such changes in G_exc_ and Ginh would not only be in opposite directions, contrary to our findings, but their magnitude would be significantly smaller compared to the blocking effects of WIN55212-2 on synaptic conductances that we found (G_exc_ = 39 ± 15% and G_inh_ = 23 ± 17%). Moreover, no significant labeling for NKCC1 has been detected in the IPL and the ganglion cell layer in the adult mouse retina (Li et al., 2008). Thus, this discrepancy in the effect of cannabinoid receptor activation on membrane potential and cell excitability may be due to different mechanisms of action operating in these two species. The WIN55212-2-induced reduction of the inhibitory conductance can be explained by a direct effect on amacrine cells as well as indirectly due to a reduction of the excitatory inputs that drive feedforward inhibition from amacrine cells onto ON-RGCs (Murphy and Rieke, 2006). The mechanism underlying the spatial modulation of the receptive field profile by cannabinoids seems to involve fine tuning of the strength of these two neurotransmitters of opposing effects in different synapses. WIN55212-2 decreased the degree of surround inhibition of excitatory but not of inhibitory inputs. This decrease in surround inhibition of the excitatory conductance can be explained if the excitatory inputs that drive the amacrine cells that provide presynaptic inhibition to ON bipolar cells are stronger than those involved in feedforward inhibition or if cannabinoid receptors are differentially distributed in these subsets of amacrine cells. Signal transmission through such networks, however, is the most parsimonious explanation as RGCs integrate inputs that are the result of signal processing in the inner and outer plexiform layers and most synapses involved in retinal processing can potentially be modulated by cannabinoids (Bouskila et al., 2016). Further research using genetic labeling, cell ablation and/or optogenetic techniques such as those employed in recent studies of the retinal circuit to target specific bipolar and amacrine cell types (Lee et al., 2011; Jacoby et al., 2015; Tang et al., 2015; Tien et al., 2016) may help dissect more precisely the target/s of (endo) cannabinoids in the signaling pathways to ON-S RGCs.

The CB1R inverse agonist AM251 caused a significant increase in the peak spike response and in surround inhibition demonstrating that, in dark-adapted conditions, ON-S RGCs are modulated by endocannabinoids. Given the non-linear relationship between amplitude of postsynaptic potentials and spike response, the increase in amplitude of the light-evoked postsynaptic potential, although it was found to be non-significant, is likely to be responsible for the significant increase in peak spike response. Conductance analysis showed that the magnitude of the peak excitatory conductance was increased to a greater extent than that of the peak inhibitory conductance. Whilst the increase in excitation indicates a stronger bipolar cell output, the increase in inhibition may originate from a direct effect on presynaptic cannabinoid receptors in amacrine cells and/or a stronger excitatory drive to amacrine cells that provide feedforward inhibition. In the presence of AM251 the inhibitory conductance was less inhibited by surround stimulation, leading to stronger inhibitory inputs from the surround which in turn would cause a sharper tuning of the spike response. This increase in the strength of inhibitory inputs under surround illumination is likely to arise from an increase in the strength of the excitatory inputs that drive inhibitory amacrine cells that provide direct inhibition to ON-S RGCs. Surprisingly, the spatial tuning of the excitatory conductance was not modified by AM251 as would be expected from its enhancing effect on the magnitude of inhibition in response to surround stimulation. This, as previously discussed, suggests that amacrine cells providing presynaptic inhibition to the bipolar cells that drive ON-S RGCs are likely to be different from those involved in feedforward inhibition, therefore a reduction in excitation will impact these forms of inhibition in different ways.

The receptive field of RGCs at high luminance levels are sharply tuned and have a strong inhibitory surround whilst at low luminance levels their spatial tuning is broader as a consequence of weaker inhibitory surround mechanisms (Derrington and Lennie, 1982; Shapley and Enroth-Cugell, 1984; Farrow et al., 2013). Studies on amphibian and fish retina suggested that the cannabinoid system is involved in modulating retinal sensitivity under different luminance conditions, in dark and/or light adaptation and in contrast adaptation (Fan and Yazulla, 2004, 2005; Yazulla, 2008; Miraucourt et al., 2016). Moreover, cannabinoids were shown to have an antagonistic effect to dopamine, a transmitter involved in the switch from rod to cone mediated vision, in cones and bipolar cells of the goldfish retina (Fan and Yazulla, 2005) and in the mammalian retina cannabinoids were shown to suppress dopamine release (Schlicker et al., 1996; Weber and Schlicker, 2001). Our findings, that activation of cannabinoid receptors reduces response strength and surround inhibition and that reduction of endocannabinoid activity leads to stronger responses and sharper spatial tuning in ON-S RGCs, are consistent with the luminance-dependent changes in gain control and receptive field properties of RGCs and with the postulated role of cannabinoids as a “dark signal” (Yazulla, 2008; Farrow et al., 2013). It remains to be elucidated, however, under what luminance and physiological conditions the cannabinoid system is active in the retina and whether or not the eCB system is involved in plasticity phenomena such as contrast adaptation or dark-adaptation in the retina.

Morphological and functional expression of the endocannabinoid system has also been demonstrated in other areas of the visual pathway, such as the dorsal lateral geniculate nucleus (dLGN) and visual cortices (V1 and V2) in rodents and primates (Eggan and Lewis, 2006; Dasilva et al., 2012; Yoneda et al., 2013; Abbas Farishta et al., 2015; Javadi et al., 2015). Visual-evoked responses from rat dLGN neurons show two populations of cells that respond differentially to cannabinoids: the majority (72%) of dLGN neurons are inhibited by cannabinoid agonists, an effect that is prevented by AM251, whilst the remaining 28% are stimulated (Dasilva et al., 2012). At the level of visual cortex, the role of the endocannabinoid system in the development of GABAergic neurotransmission (Jiang et al., 2010) and ocular dominance plasticity (Liu et al., 2008) in rodents is very well documented. Cannabinoid agonists have also been reported to modulate visual responses in the primate primary and secondary visual cortices by decreasing EEG power, LFP power and coherence whereas single cell responses show modulation of their temporal dynamics (Ohiorhenuan et al., 2014). This suggests that the cannabinoid system exerts its effects at different levels of the visual system. Although a more systematic study of the effect of cannabinoids in different types of RGCs and other neurons in the different areas of the visual pathway is necessary, the overall effects at different stages of visual processing seem to be a consistent cannabinoid-induced reduction of visual responses.

Taken together, our results demonstrate the functional expression of the retinal cannabinoid system and show how activation of cannabinoid receptors modifies the response strength and the spatial tuning properties of ON-S RGCs to acquire properties characteristic of low light level conditions. This suggests that alterations, either pathological or induced by exocannabinoids, in the cannabinergic system, might have profound effects on the transmission of light signals and consequently in vision.

## Materials and Methods

### Tissue Preparation

Dark-adapted adult C57Bl/6J mice (>6 weeks) of either sex were anesthetized with isoflurane and euthanized by cervical dislocation in the dark. Tissue dissection was performed in the dark under infrared light using infrared night viewers (FJW optical systems) to maintain the dark-adapted state of the retina. Recordings were performed in darkness. The eyes were enucleated and dissected in carboxygenated AMES medium. The cornea, iris, and vitreous were subsequently removed and the retina was detached from the sclera. A hemiretina was mounted photoreceptor side down in a recording chamber, which was then transferred to an upright microscope and observed via a CCD camera under infrared illumination (Axioskop 40, Zeiss). The tissue was continually perfused with carboxygenated AMES medium at 3–5 ml/min at 35°C. A small hole was torn in the inner limiting membrane with an empty patch pipette to gain access to the ganglion cell layer.

### Recordings

Whole-cell patch-clamp recordings were obtained using borosilicate glass pipettes with a resistance of 6–8 MΩ. High resistance seals (>1 GΩ) were made on the cell body of large neurons (>18 μm diameter) in the ganglion cell layer. Recordings were made in both current-clamp and voltage-clamp configurations. Responses were recorded using Pulse 8.67 (HEKA Electronik) software.

### Solutions

Current-clamp recordings were obtained with an internal solution containing (in mM) K-Glu: 140, HEPES: 10, EGTA: 10, MgCl_2_: 4.6, ATP-Na: 4. GTP-Na: 0.5 and 2% Lucifer yellow (LY) for cell identification. Voltage-clamp recordings were obtained with an internal solution that contained (in mM) Caesium Methanesulphonate: 100, HEPES: 20, EGTA: 10, CaCl2: 1, MgCl2: 4.6, ATP-Na: 4, GTP-Na: 0.4, creatine phosphate: 20, creatine phosphokinase: 250, TBA: 5, QX-314: 5, 2% LY. Conductance analysis was done as in Taylor and Vaney (2002) and Protti et al. (2014).

Light-evoked responses were recorded before and 5 min after bath application of a cannabinoid receptor (CB1R/CB2R) agonist (WIN55212-2; 10 μM) or a CB1R inverse agonist (AM251; 5 μM). Stock solutions of WIN55212-2 and AM251 were prepared in DMSO (DMSO concentration in Ames solution was maintained at <0.1%). All chemicals and drugs were obtained from Sigma except for Ames that was purchased from United States Biological.

### Visual Stimuli and Recording Protocols

Retinal tissue was continuously exposed to a background of 0.025 cd/m^2^ mean luminance (mesopic conditions). Visual stimuli were focused on the photoreceptor layer of the retina through the microscope optics using a DLP projector (Infocus LP120, 60 Hz). Spontaneous spike rate was determined by a 3 min continuous recording on background luminance. For current-clamp experiments, stimuli consisted of bright uniform circular spots of varying diameters (10 different sizes between 77 and 1400 μm). Stimuli were presented for 500 ms on a background (mean luminance, L_b_ = 0.025 cd/m^2^).

Voltage-clamp experiments were conducted to estimate synaptic conductances using spots of 5 different diameters (84, 266, 425, 851, and 1275 μm). Spots of increasing (bright) or decreasing (dark) luminance were presented for 500 ms on a background (luminance L_b_ = 0.025 cd/m^2^). The intensity of bright and dark stimuli (L_stim_) was adjusted to +99 and -99% of their Weber contrast (L_stim_-L_b_/L_b_), respectively. The sequence of stimuli was randomized to remove any time-based or cell stress-based bias. Stimuli were generated using the visual stimulus generating software EXPO (P. Lennie, University of Rochester, Rochester, NY, United States).

### Data Analysis

Data analyses were carried out using custom written routines in Igor Pro (Wavemetrics, Lake Oswego, OR, United States).

#### Spike Detection

Traces were analyzed to determine spontaneous spike frequency and stimulus-evoked spike responses using custom written routines. Action potentials were detected by using an off-line routine to locate the maxima by calculating the smooth first and second derivative of the voltage signal and comparing it to a threshold typically set between -35 and -30 mV.

#### Area–Response Function

The spatial organization of receptive fields was analyzed by measuring area–response functions from spike counts of visual-evoked responses. The average of two stimulus presentations for each size was used to calculate the final spike output and was then plotted against stimulus diameter (spontaneous spike rate was subtracted before analysis). The maximum value from the area-response function represents the “peak response,” which is an indicator of the receptive field center size. A “suppression index” (SI) was determined to quantify the reduction caused by the antagonistic surround of the receptive field. SI was calculated using the following formula:

$$SI=\left(1-\left(R_{\max}/R_{peak}\right)\right)\times 100$$

where R_peak_ is the peak response and R_max_ is the light-evoked response obtained using the largest spot size that stimulates both the center and surround of the receptive field. The data was fitted to a Difference of Gaussians (DoG) model of the receptive field (Rodieck, 1965; Rodieck and Stone, 1965; Enroth-Cugell and Robson, 1966) which assumes the receptive field consists of a narrow Gaussian representing the center and a broader Gaussian of opposite polarity representing the surround:

$$R(s)=K_{C}\times erf{\left(s/d_{C}\right)}^{2}-K_{S}\times erf{\left(s/d_{S}\right)}^{2}$$

Where s is the diameter of the stimulus, erf is the error function, K_c_ and d_c_ are the strength and diameter of the center, and K_s_ and d_s_ are the strength and diameter of the surround, respectively. Response strength and spot sizes were normalized to optimal spot size to allow comparisons of the receptive field properties of cells recorded at different eccentricities, a procedure previously used to characterize the receptive field surround of bipolar cells (Eggers and Lukasiewicz, 2010) and RGCs (Sagdullaev and McCall, 2005; Protti et al., 2014).

### Conductance Analysis

Excitatory and inhibitory synaptic inputs to the RGCs were measured by extracting synaptic conductances using a modified version of previously described methods (Borg-Graham, 2001; Taylor and Vaney, 2002; Di Marco et al., 2009). Light evoked responses for each spot size were recorded at 6 holding potentials (from -100 to +25 mV in 25 mV steps). Six traces consisting of current-to-voltage relationships for each stimulus size and contrast were extracted for analysis. Excitatory and inhibitory synaptic conductances were then obtained from these recordings by performing a least-squares fit to synaptic I-V relations using the equation:

$$I(V_{m})=G_{inh}(V_{m}-E_{{Cl}^{-}})+G_{exc}\left(V_{m}-V_{exc}\right)$$

Where V_m_ is membrane potential, G_inh_ is the inhibitory conductance, E_Cl_^-^ is the chloride reversal potential (estimated to be -65 mV), G_exc_ is the excitatory conductance and V_exc_ is the excitatory reversal potential (estimated to be 0 mV). Total conductance: G_T_ and reversal potential: V_rev_ were estimated and used to generate G_exc_ and G_inh_ using these formulae:

$$G_{exc}(t)=\frac{G_{T}(t)\times \left[V_{rev}(t)-E_{{Cl}^{-}}\right]}{V_{exc}-E_{{Cl}^{-}}}$$

$$G_{inh}(t)=\frac{G_{T}(t)\times \left[V_{rev}(t)-V_{exc}\right]}{E_{{Cl}^{-}}-V_{exc}}$$

The magnitude of excitatory and inhibitory synaptic inputs was quantified as the integral of their conductances. The DoG function for each cells conductance was normalized to the G_exc_ maximum for each cell then combined with other cells with similar response behavior to generate an average conductance trace. These traces were smoothed using a three-point average and overlaid onto an error trace using ± SEM.

### Morphological Classification of RGCs

Cell morphology was revealed after completion of electrophysiological recordings, by exposing the fluorescent labeling of the soma and dendritic tree for digital image capture. The tissue was subsequently fixed in paraformaldehyde (4%) for 1 h, washed out with PBS (phosphate buffered saline 0.2M). Fixed retinae were incubated with an antibody against LY (rabbit IgG, 1:10000, Invitrogen) for 16 h, and then incubated with Alexa 594 conjugated to goat anti-rabbit IgG (1:500, Invitrogen) for 5 days. Digital images were taken with a mercury lamp to allow further morphological classification. Cells were identified based on the description (branching type and density) and parameters (soma and dendritic field size) provided in the most recent morphological survey of mouse ON-α RGCs (Bleckert et al., 2014). The average soma size of the population of cells we recorded from was 20.1 ± 2.5 μm (range 16–24 μm) and their average dendritic tree diameter was 324 ± 64 μm (range 235–447 μm), values within the range described by Bleckert et al. (2014). Throughout the recordings cells displayed sustained action potential firing in current-clamp mode in response to increases in light-intensity and conductance analysis revealed their light-evoked synaptic inputs as identical to those described by Murphy and Rieke (2006) and van Wyk et al. (2009) for ON-α cells.

### Statistical Analysis

Normality of the data was assessed using the D’Agostino-Pearson omnibus K2 test for datasets containing 8 or more values whilst the Shapiro–Wilk test was used for datasets with less than 8 values. For data displaying normal distribution, Student’s *t*-test was used to test the equality or difference of mean values. All analyses were tested for *p* < 0.05 significance levels. Statistical analyses were conducted with GraphPad Prism 7. All error bars are ± SEM except for Figure 1D, 2D where they represent standard deviation.

## Data Availability

The datasets generated for this study are available on request to the corresponding author.

## Ethics Statement

Procedures were approved by institutional (The University of Sydney) Animal Ethics Committee, and conform to both the Society for Neuroscience and National Health and Medical Research Council of Australia policies on the use of animals in research.

## Author Contributions

DP designed the research. TM and JH performed the research. TM and DP analyzed the data and wrote the manuscript.

## Conflict of Interest Statement

The authors declare that the research was conducted in the absence of any commercial or financial relationships that could be construed as a potential conflict of interest.
